# Modern Sociology

**Published:** 1899-07-15

**Authors:** 


### Modern Sociology.

### PLAY-ACTING A SUBSTITUTE FOR SERMONS.

We all like to treat our boys and girls to Christmas pantomime, and we all know what a deep and enduring impression is made on the little ones by the first plays they are taken to see. It seems, however, impossible to impress the educators of the nation with the importance of these data.

Many of us remember the time when theatres were generally still talked down in accordance with the great Puritan wave which submerged us as a nation for so many generations. To-day a happier tone prevails; but yet our theatre is looked upon as recreation after the meritorious fulfilment of the duties of our state. We do not look on theatre-going as a duty. Think what an immense advance on our standpoint has been gained in Denmark, where the State theatre is a recognised educating apparatus. Even in Germany it has been the custom for years to perform standard historical plays on a given day in the week, to which all those in *statu pupillari* are entitled to seats at half price. Who has not been struck by the enterprise of Swiss schoolmasters marshalling small armies of pupils to free performances of "William Tell"? The theatre interests all our faculties of thought, judgment, and imagination. The impression made even on the fully developed is often overwhelming, and yet we allow the minds of children to be bored by long-winded verbal dissertations, instead of sending them to the stimulating influence of wholesome and well acted plays.

In the religious world the Roman Catholic Church has ever been keenly alive to dramatic effect; those of other creeds cannot withstand the æsthetic influences of that stately and well-ordered ritual. It is curious to observe the eager faces directed to the Palm Sunday procession and to note the long enduring patience of crowds who stand on the stone flags of the Brompton Oratory, crowds who would never be brought to fill the luxurious pews of other churches. Here there is a bond of sympathy between the crowd and the procession, and the reason of this is not far to seek; the people understand what they see with their eyes. Few of us are made for abstract thought, and for the vast majority seeing goes a long way towards believing. It has been a growing custom among us to go to the religious plays handed down by tradition at Oberammergau—we come away edified and refreshed, having drunk in a store of vigorous emotion, yet those who go to these plays write, for the most part, against the revival of mystery plays in England. This is precisely the opinion we would wish to combat. We hold that it is advisable to cause the events upon which the Christian creed rests to be dramatised under proper supervision and at suitable hours in churches. We would even go so far as to suggest the reconstruction of the various confraternities of actors as part of the parochial machinery. That for which we will not exert ourselves is non-existent for us. It is easy to trace the wonderful success of the mission at St. Alban's, Holborn, to the stress laid by the clergy on action. A young man is made to feel his importance to the community, he not only goes to the mission, he is the mission, and if he is made responsible for the mere carrying of a banner he is there in his place punctual and collected. Responsibility is the mother of enthusiasm. We have heard of young men who walked on a Sunday morning from Chislehurst to Holborn rather than forego the privilege of marching in the procession. Now, as we think, a procession is essentially a form of drama. It is a convenient allegorical representation of the Church, with its clergy of varying ranks, its laity, its seasons of triumph or woe, and, like the drama, the procession appeals to an audience which appreciates and sympathises, and takes its impressions and sympathies away out of church into the humdrum life of the great metropolis. The greater the impression on the individual the greater the temporary self-forgetfulness produced, the more does that individual benefit; and if this be so in the case of a simple procession it would be still more so were the procession elaborated from antiphon and chorus into a simple drama with suitable scenes and dresses.

We are much against the introduction of religious subjects into the secular drama. Yet a play like "The Sign of the Cross," with its unpardonable lapses into melodrama, and with its æsthetically barbarous action, has proved both in London and in the provinces a financial success, while many another play of greater merit has ruined its company. From this we argue a predisposition in the bulk of the people to like a dramatic representation of that which their mothers have taught them. If such representations were given on Sunday afternoons the result would be electrifying. Sunday concerts have been a success, and Sunday plays, in dignified surroundings, as in the Abbey, in St. Paul's, and in the other great churches, with simple dignified ritual and accompanied by slow and solemn music, would revolutionise our religious ideals. To quote Homer, Iliad, 18—54, "Thus with these eyes I behold, and my heart is troubled within me."

Six days of school and one day free would be really the lot of our fortunate children, while their great ambition will be to act or help in the Sunday play. The impetus to amateur solos, to chorus singing and recitation would be simply immense. The lost art of reading aloud would be recovered, clumsiness and awkwardness of gait and manner would be cured, while that painful self-consciousness—so at variance with the discharge of social duties—would be generally and speedily overcome.

